# The sublethal effects of neonicotinoids on spiders are independent of their nutritional status

**DOI:** 10.1038/s41598-021-87935-z

**Published:** 2021-04-19

**Authors:** Milan Řezáč, Nela Gloríková, Shawn M. Wilder, Petr Heneberg

**Affiliations:** 1 Crop Research Institute, Prague, Czech Republic; 2 Department of Integrative Biology, Oklahoma State University, Stillwater, OK USA; 3 Third Faculty of Medicine, Charles University, Ruská 87, 100 00 Prague, Czech Republic

**Keywords:** Entomology, Fat metabolism, Agroecology

## Abstract

Spiders were recently shown to be adversely affected by field-realistic concentrations of a broad scale of neonicotinoid insecticides. Among the reported effects of neonicotinoids on invertebrates were declines in lipid biosynthesis and upregulation of β-oxidation, while vertebrate models suggest increased adipogenesis following treatment with neonicotinoids. Therefore, we hypothesized that there exists synergy between the effects of diet and concurrent exposure to field-realistic concentrations of neonicotinoid insecticides. To address this hypothesis, we fed first instars of the large wolf spider *Hogna antelucana* with two types of diets and exposed them to field-realistic concentrations of three formulations of neonicotinoids (thiamethoxam, thiacloprid and acetamiprid). We then measured the growth of the tested spiders; the lipid and protein content of their bodies; and their behavior, including ballooning, rappelling, and locomotor parameters. The two tested diets consisted of casein-treated and sucrose-treated *Drosophila melanogaster*. The dietary treatments affected the lipid and protein content of the spiders, their body weight and carapace length but did not affect any of the measured behavioral parameters. Surprisingly, we did not find any effects of acute exposure to neonicotinoid insecticides on the lipid or protein reserves of spiders. Exposure to neonicotinoids altered the behavior of the spiders as reported previously in other spider species; however, these effects were not affected by dietary treatments. Overall, the dietary treatments did not have any major synergy with acute exposure to field-realistic concentrations of neonicotinoid insecticides.

## Introduction

The total quantities or balance of nutrients in the diet may modulate the effects of toxic compounds, including heavy metals; polycyclic aromatic hydrocarbons; polychlorinated biphenyls^1^; and pesticides^2,3^, including neonicotinoid insecticides^4,5^. Synergistic effects of multiple stressors have been previously documented, including synergies between nutritional stress and the effects of pesticides^6^. For example, the effects of certain pesticides on bees are increased when flower availability is restricted^7^. However, experimental studies are lacking for most diet-pesticide combinations.

The effects of dietary imbalance have been studied extensively in herbivores and omnivores, but fewer studies have examined these effects in predators^8,9^. Predators, especially invertebrates such as spiders, have repeatedly been shown to have food-limited population sizes in nature^10–12^; as a result, they have evolved physiological adaptations to allow them to adjust their metabolism to food availability^13–17^. Moreover, recently published data suggest that diet affects various aspects of spider behavior^18,19^; therefore, there is a potential for synergistic, additive or potentiating effects of combined exposure to altered diet and exposure to compounds known to affect spider behavior, such as neonicotinoid insecticides^20,21^. In nature, prey may vary widely in nutritional content both within and among species (e.g., 5–30% of lipids and 20–80% of proteins)^22–25^. Some studies of spiders have identified positive effects of protein-rich diets on carapace length and positive effects of lipid-rich diets on body weight^25–29^. Accelerated growth and improved survival of spiders were reported by multiple studies using varied diets or nutrient-supplemented prey^27,30–34^. Other studies found that spiders assimilated only small and relatively steady amounts of nutrients when fed prey of various nutrient content^35^ and regulated their lipid and protein intake when possible^33,36,37^.

Studies of predator nutrition have been hindered by the difficulties of directly and independently manipulating multiple nutrients in prey in a controlled way^38^. To overcome this challenge, some studies provided spiders with prey whose lipid and lipoprotein content had been artificially increased, using various methods including injecting lipids directly into the prey and feeding the prey animals with diets that increased their lipid content. Caution is needed with experiments on artificially added lipids, such as the linoleic acid, linolenic acids and cholesterol, although linoleic acid is an essential fatty acid for spiders^34^. The apparent absence of effects of these additives on spider growth^39^ could likely be overcome by the use of conjugated fatty acids instead of their cis-9,cis-12 (linoleic) and cis,cis,cis-9,12,15 (linolenic) isomers, as the latter isomers lack the strong biological activities that are known to be associated with conjugated fatty acids^40–42^. Increasing the lipid content of prey can result in a reduction, no change or an increase in the growth of juvenile spiders, depending on the species^29,33,34^. However, there are consistent increases in the lipid content of spiders when they are fed on high-lipid prey^29,33,34,43^. Spiders also increase their consumption of low-protein foods to maintain their intake of lipids and energy^28^.

Neonicotinoid insecticides are well known to have negative effects on the diversity of invertebrates, particularly pollinators^44–48^. Spiders are less affected by neonicotinoids than bees are, owing to differences in the structure of acetylcholine receptors that mediate the action of neonicotinoids in invertebrates^49^. Regardless, neonicotinoids are partially lethal to spiders in field-realistic concentrations, inducing also temporary paralysis, impairing the structure of spider silk and affecting spider chemoreception^20,21,50,51^. Moreover, neonicotinoids deter feeding and therefore negatively affect the food consumption rates and likely the nutritional status of spiders^52^. Concerning the relationship between dietary lipids and neonicotinoid insecticides, data from spiders are lacking. However, relevant studies have been performed in mammalian models. In mammalian models, neonicotinoids themselves promote adipogenesis and cause insulin resistance^53,54^. When a neonicotinoid insecticide (imidacloprid) was administered to mice fed a low-fat or high-fat diet, imidacloprid administration in combination with a high-fat diet facilitated body weight gain and adiposity, impaired glucose metabolism and affected AMP-activated protein kinase-α signaling (a highly conserved protein that is crucial in the maintenance of cellular energy homeostasis, including fatty acid uptake and oxidation)^55^.

In the present study, we used a model invertebrate predator, the large wolf spider *Hogna antelucana* (Montgomery, 1904) (Araneae: Lycosidae), which is widely distributed across North America^56–58^. Based on previous observations of the effects of a high-fat diet^19^ and neonicotinoids^20^ on spiders, we hypothesized the existence of synergy between the effects of diet and concurrent exposure to field-realistic concentrations of neonicotinoid insecticides. To address this hypothesis, we fed first-instar *H. antelucana* with two types of diets and exposed them to field-realistic concentrations of three formulations of neonicotinoids. We then measured the growth of the tested spiders; the lipid and protein content in their bodies; and their behavior, including ballooning, rappelling, and locomotor parameters.

## Materials and methods

### Model organism

As a model organism, we used first instars of *H. antelucana*. We collected them from the abdomens of their mothers on late summer nights on cultivated grasslands in the suburbs of Stillwater, Oklahoma, USA (36.11°N, 97.11°W). We placed the juveniles individually into vials with carbon plaster on the bottoms and enclosed them with foam plugs. This species is an abundant predator in the grasslands of the Southern USA. Wolf spiders readily exhbit ballooning behavior and silk production. We collected the study individuals for two weeks prior to the experiment. We acclimated them for at least a week in controlled conditions at 22 °C and 80% humidity with a natural light/dark cycle. In total, we tested 121 spiders; each treatment included a group of 14–16 individuals.

### Diet treatments

Spiders received five female flies twice a week and had access to water ad libitum. We manipulated the macronutrient content of the live prey items, which were flightless mutants of *Drosophila melanogaster* Meigen, 1830, by raising them on media with different macronutrient compositions such that we could perform treatments with two types of flies differing in lipid:protein ratio sensu Jensen et al.^33^ All the diets were based on Carolina Biological fly medium (Formula 4–24 Instant *Drosophila* Medium). We added bovine milk casein to the media at a ratio of 3:2 (w/w) casein:medium to increase the protein content of the resulting flies. Alternatively, we added sucrose at a ratio of 1:2 sucrose:medium to increase the lipid content of the flies. Flies that were grown on these media differed in lipid:protein ratios. New cultures of flies were started weekly^28^. The treatment lasted 26 days.

### Tested neonicotinoids

We tested the effects of three neonicotinoids (thiamethoxam, acetamiprid and thiacloprid) in formulations and concentrations that are commonly sprayed on crops to eliminate pest insects. Thiamethoxam was formulated as Actara 25 WG (Syngenta Crop Protection, Basel, Switzerland), which contained 25% of the active ingredient and had a suggested application rate of 70–80 ml ha^−1^. Thiacloprid was formulated as Biscaya 240 OD (Bayer CropScience, Monheim, Germany), which contained 22.97% of the active ingredient and had a suggested application rate of 200–300 ml ha^−1^. Thiacloprid has already been banned in the European Union since 2020 but is still actively used in the United States (mainly on cotton and fruits) and in other countries. Acetamiprid was formulated as Mospilan 20 SP (Nippon Soda Co., Tokyo, Japan), which contained 20% of the active ingredient and had a suggested application rate of 60–250 ml ha^−1^. As a vehicle and mock control, we used distilled water. Each treatment group consisted of at least 15 individuals. We sprayed the commercial neonicotinoid formulations (or distilled water) directly onto the dorsal side of the bodies of the spiders. To apply the commercial formulations of neonicotinoids, we diluted them to the maximum recommended concentrations for their use under field conditions and sprayed them at 4.2 μl cm^−2^ using a handheld atomizer (MADomizer1, LMA; Teleflex) in precisely calibrated doses. Therefore, the applied doses were 195.2 ng cm^−2^ for Actara 25 WG, 702.72 ng cm^−2^ for Biscaya 240 OD, and 488 ng cm^−2^ for Mospilan 20 SP. We sprayed the neonicotinoids twice: once at the beginning of the 26-day-long dietary intervention period and once at the very end of that period. At the beginning of the study, we measured spider body weight and rappelling behavior. A day after the second treatment, we measured all the parameters as specified below. One hour after the second treatment, we recorded the acute mortality and tested the sublethal effects only on individuals that remained alive at that time. Two days later, we weighed the spiders and processed them to analyze their macronutrient contents.

### Nutrient analysis

We stored the samples at – 20 °C until they were analyzed. Prior to analysis, we dried the samples in an oven at 60 °C for 24 h and measured the dry weight to the nearest 0.01 mg. We used a gravimetric assay to calculate lipid content for each treatment. Briefly, after obtaining dry weight, we soaked the spiders in three sequential 24-h-long liquid chloroform baths to dissolve lipids, followed by 24 h of drying in the oven. We weighed the dried spiders again to the nearest 0.01 mg. We considered the difference between the initial and final weights to be the lipid mass. After the lipid extraction, we performed a protein content analysis using a Lowry assay modified for use in microplates and employing a protein extraction method optimized for arthropods, which included solubilizing the tissue in 0.1 M NaOH^25^.

### Measurement of growth

We assessed spider growth using two measures. First, we determined body mass at the beginning and at the end of the experiment by weighing the spiders on a scale to the nearest 0.01 mg. Second, we measured the length of the carapace. We took photographs of the carapaces using a camera attached to a dissecting microscope and analyzed them with ImageJ (NIH, Bethesda, MD) software to 0.001 mm.

### Locomotor parameters

One hour after treatment with neonicotinoids, we placed the spiders into Petri dishes that were 33 mm in diameter and videotaped for 15 min. We used the tracking software EthoVision to measure the behavior of spiders during the video-recorded trials. In particular, we compared the total distance moved (mm), the mean velocity (mm s^−1^) and the angles of rotation^59^.

### Rappelling behavior

We measured rappelling as a quantitative variable (change in the distance dropped when rappelling behavior was tested prior to and after the combined treatment with diet and neonicotinoids). One and one-half hours after acclimation and treatment with neonicotinoids, we stimulated rappelling by placing the spiders on 10 × 10 cm glass square plates that were positioned atop 45-cm-long sticks. Control spiders regularly attached their dragline (major ampullate fiber) to the glass plate with attachment discs, i.e., sticky piriform fibers. Failure to display rappelling manifests by problems with anchoring the dragline to the glass plate and/or with the production of the dragline itself. We recorded the length (distance) that the spiders descended toward the bottom of the apparatus while being secured by the dragline, which also required the dragline to be successfully anchored to the substratum. The rappelling stimulation lasted 15 min or until the rappelling was completed^20^.

### Ballooning behavior

We measured ballooning as a binary variable, i.e., as the presence/absence of this behavior at the end of the 26-day study period. Two hours after acclimation and treatment with neonicotinoids, we stimulated ballooning by simulating a slight breeze at 2 m s^−1^ with a ventilator and by placing the tested spiders in dishes with vertical wooden sticks as described by Pétillon et al.^60^. This environment stimulated the control spiders to climb to the very tops of the sticks, where they adopted a tiptoe position and produced gossamer silk from their abdomens. We recorded the ratio of animals that ballooned to animals that stayed on the bottom of the dish. The conditions to stimulate ballooning lasted 15 min or until ballooning was performed. We recorded any ballooning displayed during the 15-min evaluation period as a positive test result^20^.

### Data analysis

We analyzed the data in SigmaPlot 12.0. To test the effects of the applied treatments, we employed two-way ANOVA followed by post hoc Bonferroni`s post-tests. We tested differences in binary variables using the χ^2^ test or, if any of the categories contained fewer than five cases, Fisher’s exact test. Data are shown as the mean ± SE unless stated otherwise.

## Results

### Effects of diet and neonicotinoids on lipid and protein content

**Figure 1:**
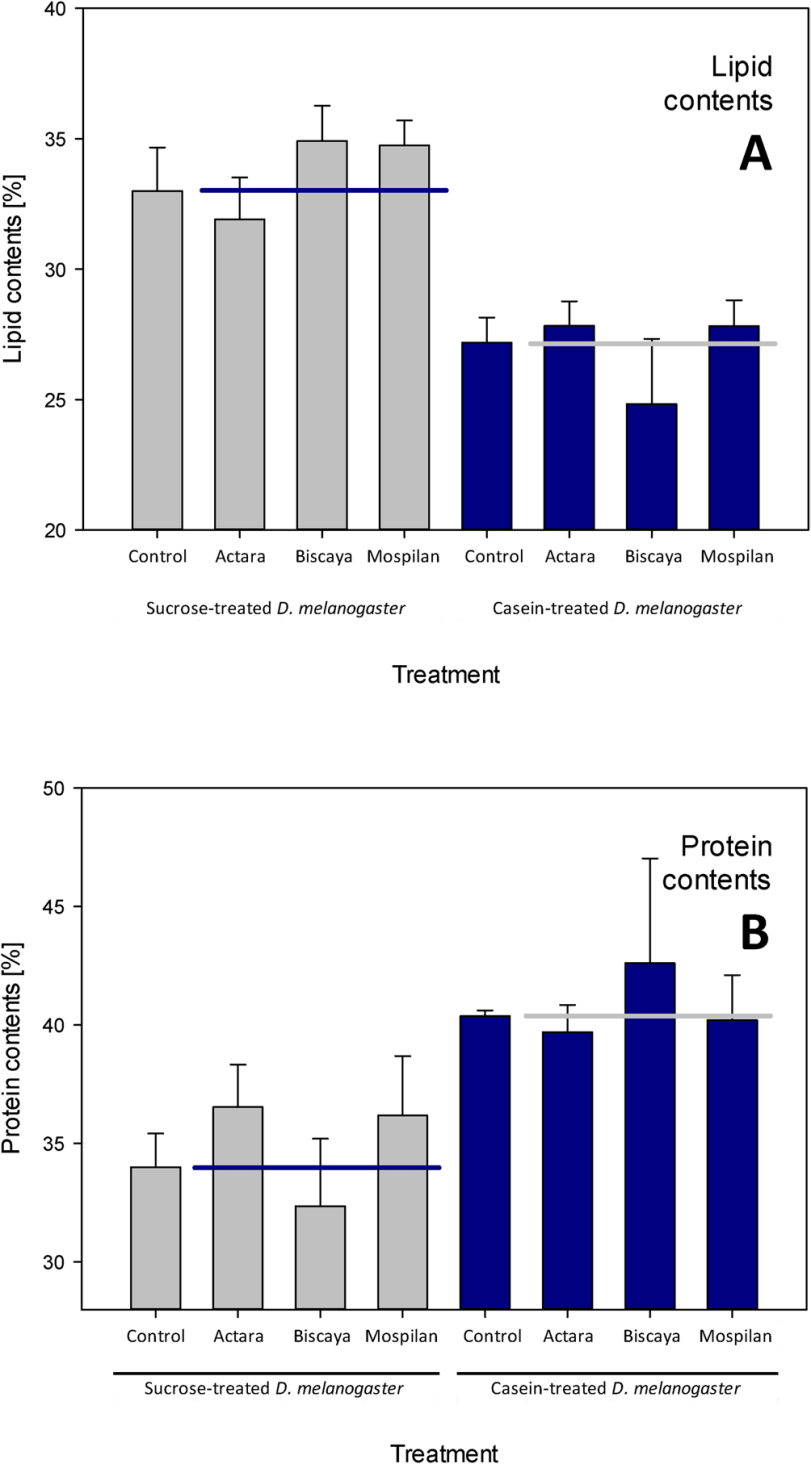
Macronutrient composition of spider bodies following dietary intervention and treatment with neonicotinoid insecticides. (**A**) Lipid contents. (**B**) Protein contents. Horizontal lines indicate mean control values.

Dietary treatment resulted in differences in the lipid and protein content of first instars of *H. antelucana*. Following a month-long treatment, the spiders fed sucrose-treated *D. melanogaster* contained 33.0 ± 1.6% of lipids (w/w of dry weight), whereas the spiders fed casein-treated *D. melanogaster* contained only 27.2 ± 0.9% of lipids. The difference in lipid content was statistically significant between the diets (two-way ANOVA F = 43.673,* p* < 0.001, df = 1). Correspondingly, the spiders fed sucrose-treated *D. melanogaster* contained less protein (40.4 ± 0.2% of proteins, w/w of dry weight), whereas the spiders fed casein-treated *D. melanogaster* contained only 34.0 ± 1.2% of proteins. The difference in protein content was also statistically significant between the two diets (two-way ANOVA F = 12.856,* p* = 0.002, df = 1) (Fig. 1).

Neonicotinoid treatments did not affect the lipid content (two-way ANOVA F = 0.445,* p* > 0.05, df = 3), and there was no interaction between diet and the administration of neonicotinoids on lipid content (two-way ANOVA F = 1.499,* p* > 0.05, df = 3). The application of the three tested formulations of neonicotinoids did not affect the protein content (two-way ANOVA F = 0.086,* p* > 0.05, df = 3), and there was no interaction between diet and the administration of neonicotinoids on the protein content (two-way ANOVA F = 0.913,* p* > 0.05, df = 3) (Fig. 1).

### Effects of diet and neonicotinoids on spider growth

**Figure 2:**
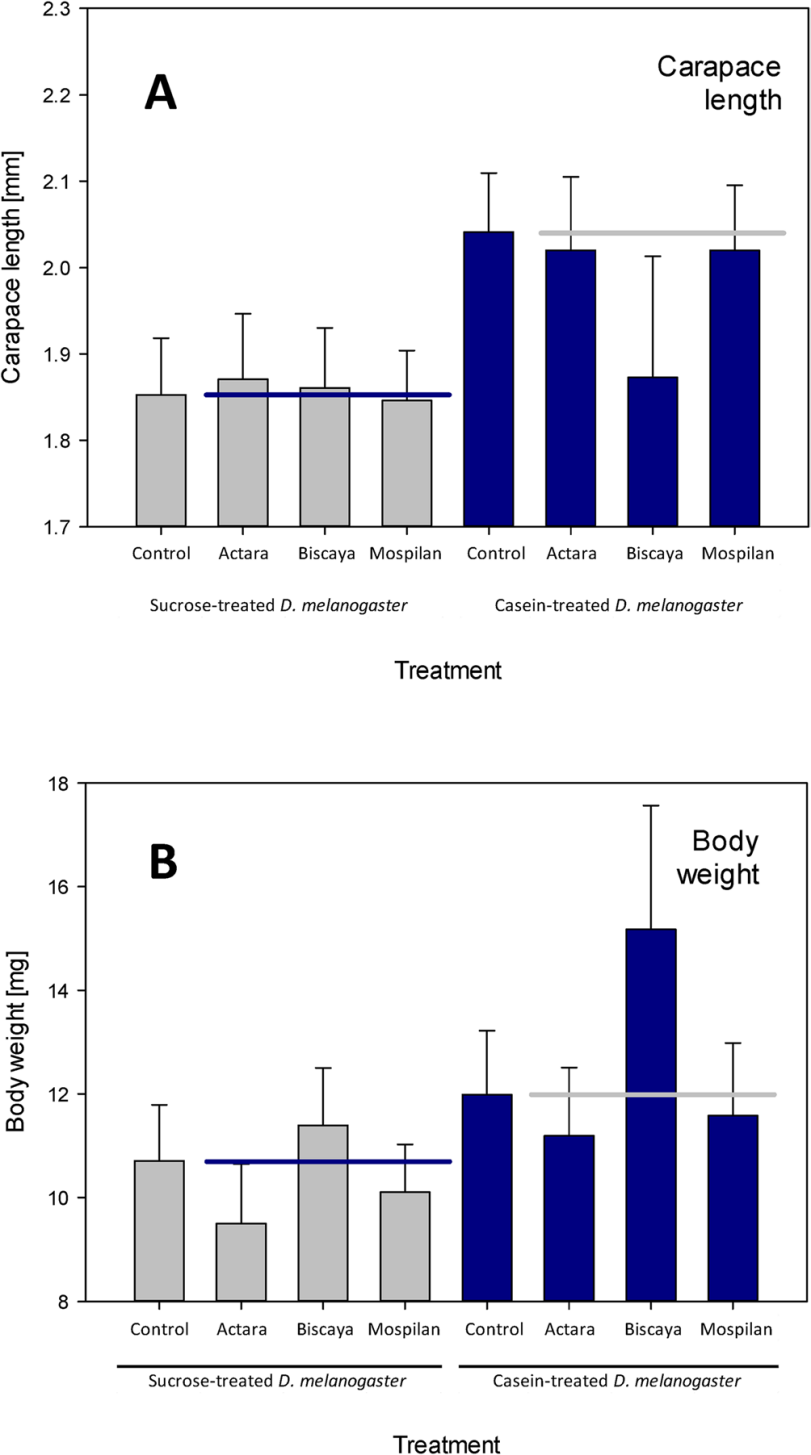
Effects of dietary intervention and treatment with neonicotinoid insecticides on spider growth. (**A**) Carapace length. (**B**) Body weight. Horizontal lines indicate mean control values.

Dietary treatment resulted reduced carapace length in spiders fed sucrose-treated *D. melanogaster* (1.853.0 ± 0.066 mm) compared to spiders fed casein-treated *D. melanogaster* (2.041 ± 0.068 mm) (two-way ANOVA F = 4.947, *p* = 0.028, df = 1). In contrast, the application of the three tested formulations of neonicotinoids did not affect carapace length (two-way ANOVA F = 0.418,* p* > 0.05, df = 3), and the effects of diet did not depend on the neonicotinoid treatment (two-way ANOVA F = 0.473, *p* > 0.05, df = 3) (Fig. 2A).

There was also a significant effect of dietary treatment on the total weight of spiders. Following a month-long treatment, the spiders fed sucrose-treated *D. melanogaster* weighed 10.7 ± 1.1 mg, whereas the spiders fed casein-treated *D. melanogaster* were slightly heavier, with a mean weight of 12.0 ± 1.2 mg (two-way ANOVA F = 4.723,* p* = 0.03, df = 1). The analysis included only spiders that survived until the end of the study period. Correspondingly, the weight gain of spiders fed sucrose-treated *D. melanogaster* (2.6 ± 0.7 mg) was slightly lower than that of spiders fed casein-treated *D. melanogaster* (4.3 ± 1.0 mg) (two-way ANOVA F = 4.638,* p* = 0.03, df = 1). The analysis included only spiders that survived until the end of the study period (Fig. 2B).

Neonicotinoid treatments did not affect the weight of spiders (two-way ANOVA F = 1.656,* p* > 0.05, df = 3), and there was no additive effect of food composition and the administration of neonicotinoids on spider weight (two-way ANOVA F = 0.343,* p* > 0.05, df = 3). Correspondingly, the application of the three tested formulations of neonicotinoids did not affect weight gain in the spiders (two-way ANOVA F = 0.666,* p* > 0.05, df = 3), and there was no additive effect of food composition and the administration of neonicotinoids on spider weight (two-way ANOVA F = 1.007,* p* > 0.05, df = 3) (Fig. 2B).

### Locomotor parameters

**Figure 3:**
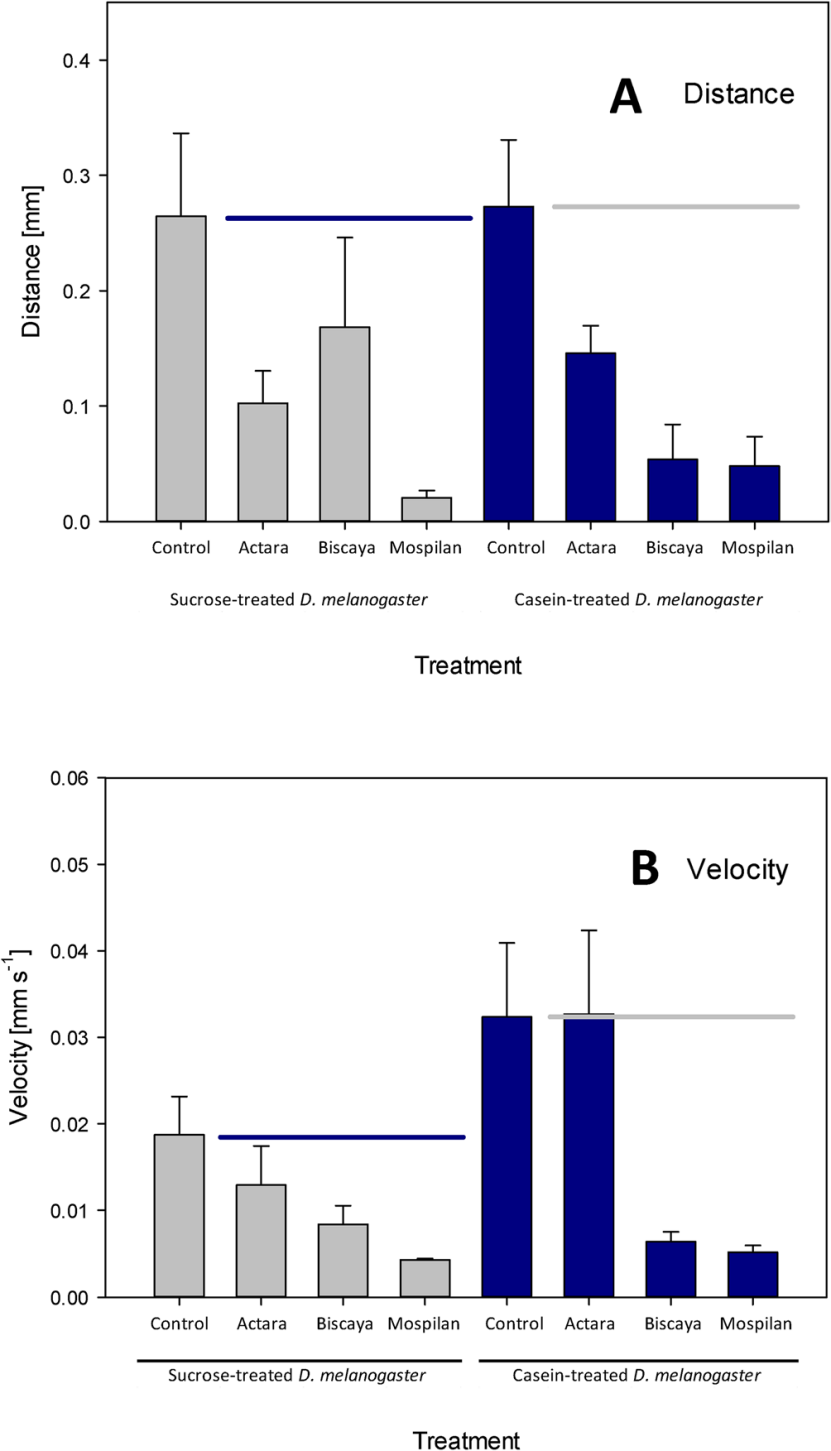
Effects of dietary intervention and treatment with neonicotinoid insecticides on locomotor parameters. (**A**) Distance moved. (**B**) Velocity. Horizontal lines indicate mean control values.

Dietary treatment did not affect the total distance moved by the spiders or their velocity. In the 15-min trials, the total travel distance of the spiders fed sucrose-treated *D. melanogaster* was 2.65 ± 0.72 mm, whereas the total travel distance of the spiders fed casein-treated *D. melanogaster* was 2.73 ± 0.58 mm (two-way ANOVA F = 0.063, *p* > 0.05, df = 1). The spiders fed sucrose-treated *D. melanogaster* had a velocity of 0.18 ± 0.04 mm s^−1^, whereas the spiders fed casein-treated *D. melanogaster* had a velocity of 0.32 ± 0.09 mm s^−1^ (two-way ANOVA F = 3.615, *p* > 0.05, df = 1) (Fig. 3).

Neonicotinoid treatments affected the total travel distance of the spiders (two-way ANOVA F = 8.431, *p* < 0.001, df = 3) but there was no interaction between diet and the administration of neonicotinoids on the distance moved (two-way ANOVA F = 0.940, *p* > 0.05, df = 3). In spiders fed sucrose-treated *D. melanogaster*, the post-tests revealed significant differences between the control group and the Mospilan-treated group (0.21 ± 0.06 mm) (Bonferroni’s post-test: diff. of means 0.244, t = 3.595, *p* = 0.003). Similarly, in spiders fed casein-treated *D. melanogaster*, the post-tests revealed significant differences between the control group and the Mospilan-treated group (0.48 ± 0.26 mm) (Bonferroni’s post-test: diff. of means 0.225, t = 3.261, *p* = 0.009) and between the control group and the Biscaya-treated group (Bonferroni`s post-test: diff. of means 0.219, t = 3.086, *p* = 0.016). The application of the three tested formulations of neonicotinoids also affected the velocity of the spiders (two-way ANOVA F = 6.408, *p* < 0.001, df = 3), but there was no additive effect of the food composition and the administration of neonicotinoids on the velocity (two-way ANOVA F = 1.447, *p* > 0.05, df = 3). In spiders fed sucrose-treated *D. melanogaster*, the post-tests did not reveal any significant differences between the control group and the treated groups. In spiders fed casein-treated *D. melanogaster*, the post-tests revealed significant differences between the control group and the Mospilan-treated group (0.052 ± 0.0079 mm s^−1^) (Bonferroni`s post-test: diff. of means 0.027, t = 3.298, *p* = 0.008) and between the control group and the Biscaya-treated group (Bonferroni’s post-tests: diff. of means 0.026, t = 3.064, *p* = 0.017) (Fig. 3).

### Rappelling and ballooning behaviors

**Figure 4:**
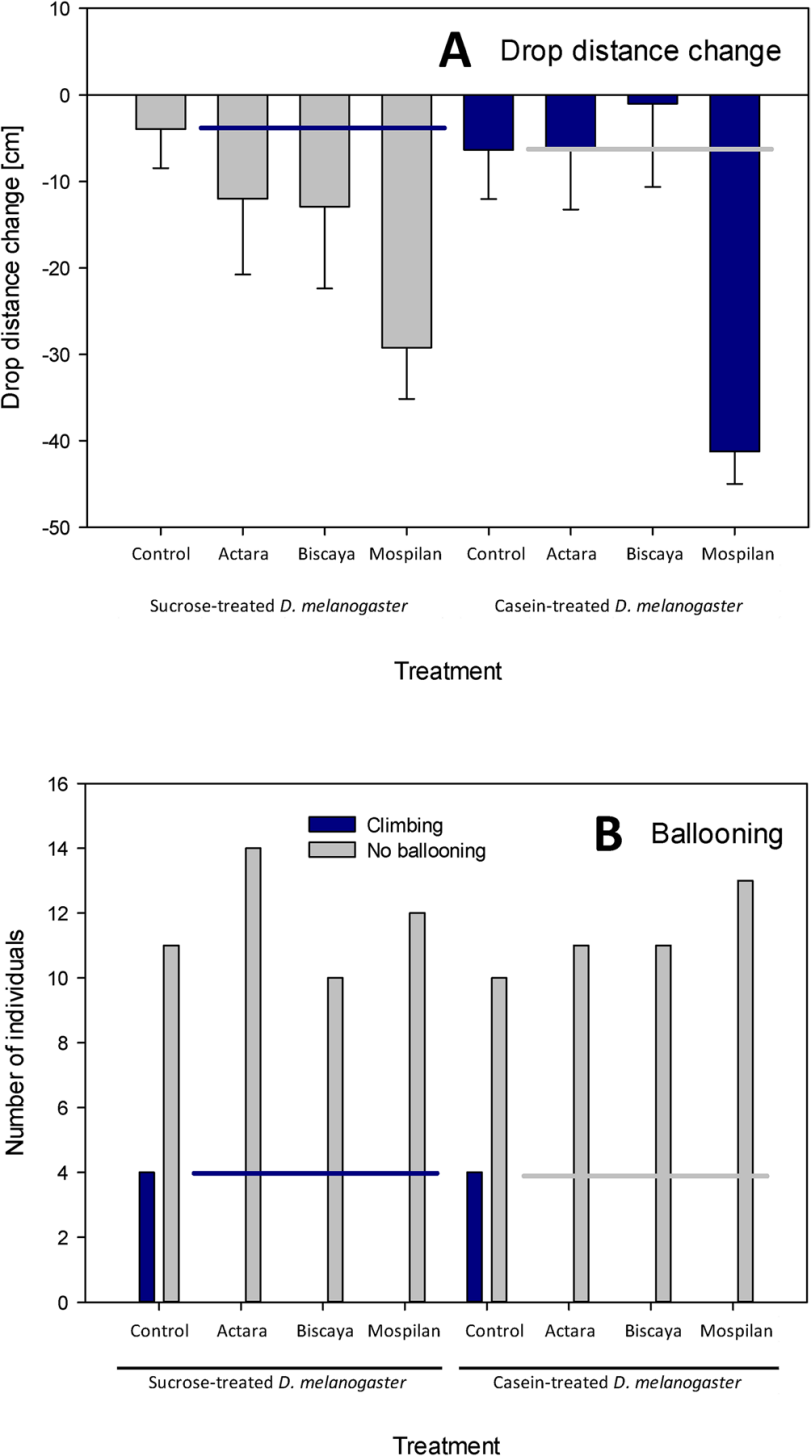
Effects of dietary intervention and treatment with neonicotinoid insecticides on rappelling and ballooning behaviors. (**A**) Change in drop distance. (**B**) Ballooning behavior. Horizontal lines indicate mean control values.

Dietary treatment did not cause a change in the total descent distance of the tested spiders. Following a month-long treatment, the change in total descent distance was − 3.9 ± 4.5 cm for the spiders fed sucrose-treated *D. melanogaster*, whereas the change in total distance dropped was − 6.33 ± 5.70 cm for the spiders fed casein-treated *D. melanogaster* (two-way ANOVA F = 0.025,* p* > 0.05, df = 1) (Fig. 4A).

Neonicotinoid treatments affected the change in the total descent distance of the spiders (two-way ANOVA F = 8.683,* p* < 0.001, df = 3) but there was no interaction between diet and the administration of neonicotinoids on the total distance dropped (two-way ANOVA F = 1.087,* p* > 0.05, df = 3). In spiders fed sucrose-treated *D. melanogaster*, the Bonferroni’s post-tests revealed significant differences between the control group and the Mospilan-treated group (− 29.2 ± 5.7 cm) (diff. of means 25.302, t = 2.723, *p* = 0.046). In spiders fed casein-treated *D. melanogaster*, the post-tests also revealed significant differences between the control group and the Mospilan-treated group (− 41.3 ± 3.8 cm) (Bonferroni’s post-tests: diff. of means 34.917, t = 3.738, *p* = 0.002) (Fig. 4A).

Concerning the presence of ballooning behavior, we observed ballooning in only four cases in each of the control groups (27% of spiders fed sucrose-treated *D. melanogaster* and 29% of spiders fed casein-treated *D. melanogaster*). Ballooning behavior was completely absent in all six groups of spiders that were treated with neonicotinoids; therefore, the tested formulations of neonicotinoids significantly suppressed ballooning behavior in the tested spiders (Fisher’s exact test *p* < 0.001) (Fig. 4B).

### Paralysis

The finding of the lack of ballooning behavior could be related to more generalized problems in the tested spiders, which could be quantified as the proportion of spiders that were completely paralyzed following the treatment with neonicotinoids and as the proportion of spiders that were not completely paralyzed but were slowed in their movements. In addition to paralysis, we also quantified the number of spiders that died.

**Figure 5:**
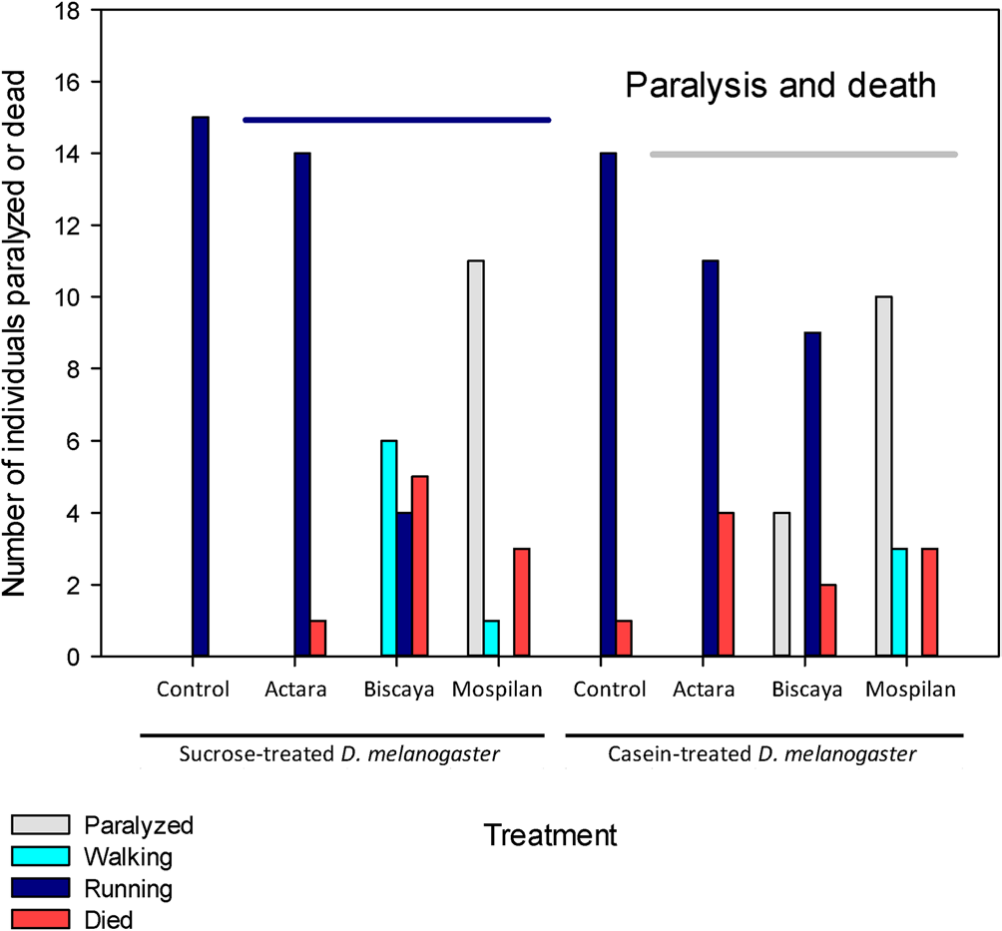
Effects of dietary intervention and treatment with neonicotinoid insecticides on the paralysis and death of the analyzed spiders. Two types of paralysis were distinguished: complete immobility (termed paralysis) and slowered movements (termed walking). Horizontal lines indicate mean control values.

As expected, the spiders fed sucrose-treated or casein-treated *D. melanogaster* did not show any signs of paralysis (no paralyzed individuals, no individuals walking instead of running). One individual in the group fed sucrose-treated *D. melanogaster* died in the course of the experiment. Treatment with neonicotinoids induced the paralysis or death of all Mospilan-treated spiders fed either sucrose-treated or casein-treated *D. melanogaster*. Among Mospilan-treated spiders, 73% of individuals fed with sucrose-treated flies and 63% of individuals fed casein-treated flies were fully paralyzed. Additionally, 7% (sucrose) and 19% (casein) of Mospilan-treated spiders showed reduced movement speed. Three individuals in each Mospilan-treated group (20% for sucrose and 19% for casein) died. The proportion of fully and only partially paralyzed Mospilan-treated spiders did not differ between those that were fed sucrose-treated and casein-treated *D. melanogaster* (Fisher’s exact test *p* > 0.05). The other two tested neonicotinoid formulations had milder effects. Actara did not induce any paralysis and was associated with 27% and 6% mortality in the spiders fed sucrose-treated and casein-treated *D. melanogaster*, respectively (Fisher’s exact test *p* > 0.05). Biscaya treatment was associated with relatively high mortality in both the sucrose and casein groups (13% and 33%, respectively) and induced full or partial paralysis in the surviving 31% and 60% of spiders, respectively (Fisher’s exact test *p* > 0.05) (Fig. 5).

## Discussion

We were unable to reproduce the results reported in a vertebrate model, where a nicotinoid promoted weight gain and adiposity^55^. We confirmed that both dietary treatment and acute exposure to field-realistic concentrations of the neonicotinoid insecticides affected the resulting phenotypes of the analyzed *H. antelucana* spiders. However, as each of the treatments affected different phenotypes, we did not find any major synergy between them. Moreover, caution is needed when attempting to generalize the observed effects. Many of the observed effects were specific to one or two of the three tested neonicotinoids.

The combination of dietary treatment and acute exposure to neonicotinoids had little effect on the body parameters of the examined spiders beyond the effect of the diet itself. The lipid and protein content of spiders were not affected by the neonicotinoids (Fig. 1); neither was the carapace length (Fig. 2A). However, some of the Biscaya-treated individuals had reduced carapace length when fed casein-treated but not sucrose-treated *D. melanogaster*; this effect was not significant, as we did not design the present study to be able to test for the specific presence of a subgroup of outliers. However, the presence of differences in the dimensions of rather uniform physical structures of spider bodies following combined treatment with casein-treated *D. melanogaster* and Biscaya requires further attention. These data are of interest, particularly in combination with the other findings, as Biscaya-treated spiders fed casein-treated *D. melanogaster* did not differ in their protein or lipid content and displayed a trend towards higher body weight than the control spiders (Fig. 2B). These findings suggest that a combination of dietary changes and chronic exposure to neonicotinoids (e.g., by means of the administration of neonicotinoid-treated prey) is needed to provide definitive data on whether there are any effects of combined treatment with casein-treated *D. melanogaster* and Biscaya. The analyzed spiders suffered from paralysis and mortality due to the administration of neonicotinoids, but these parameters were similar regardless of the dietary treatment (Fig. 5).

Concerning the locomotor parameters, acute exposure to neonicotinoids resulted in severe decreases in both the velocity of the spiders and the distance they moved. However, as the dietary intervention did not affect these locomotor parameters, we did not observe any synergy between dietary intervention and acute neonicotinoid exposure. Similarly, only the neonicotinoids affected ballooning and rappelling behaviors, whereas the dietary treatment did not have any effect either alone or in combination with the insecticides (Fig. 4).

The absence of a synergistic response to dietary intervention and acute exposure to neonicotinoids is surprising. The effects of neonicotinoids on lipogenesis have been reported in multiple groups of invertebrates. Radwan and Mohamed reported a decline in body lipids following neonicotinoid treatment of *Helix aspersa* snails^61^. In parallel to the neonicotinoid-induced decline in lipids, they observed depletion of glycogen. The decline in lipids and glycogen is actually a commonly observed pattern elicited by a catabolic response to treatment with numerous chemical compounds across multiple invertebrate taxa^62–64^. Decreased lipid content was also reported by organ-specific studies that focused on the ovaries of cockroaches^65^ and beetles^66^; those studies also found increased lipid peroxidation in neonicotinoid-treated individuals. In snails, neonicotinoid treatment downregulated lipid biosynthesis and upregulated β-oxidation of fatty acids^67^. In oysters, neonicotinoid treatment increased the contents of saturated fatty acids and altered the ratio of omega-3 to omega-6 fatty acids^68^. The effects of neonicotinoids on the protein content of invertebrates were also previously examined, with studies on snails, earthworms and terrestrial isopods, concluding that the protein content increased following neonicotinoid treatment^61,69,70^.

In conclusion, the present study found a surprising lack of effects of acute exposure to neonicotinoid insecticides on the lipid and protein reserves of spiders. Exposure to neonicotinoids altered the behavior of spiders, as reported previously in other spider species; however, these effects were not further potentiated by dietary interventions. Dietary treatment did not have any major synergistic effects with acute exposure to field-realistic concentrations of the neonicotinoid insecticides.
